# Autologous Chondrocyte Implantation With Collagen Membrane Using a Knotless Suture Bridge Technique

**DOI:** 10.7759/cureus.52568

**Published:** 2024-01-19

**Authors:** Toshihiro Seki, Kazushige Seki, Takashi Imagama, Tomoya Okazaki, Takashi Sakai

**Affiliations:** 1 Department of Orthopedic Surgery, Yamaguchi University Graduate School of Medicine, Ube, JPN

**Keywords:** bioabsorbable anchor, suture bridge technique, biological cartilage repair, chondral lesion, collagen membrane, aci, autologous chondrocyte implantation

## Abstract

Autologous chondrocyte implantation (ACI) has been covered by insurance in Japan since April 2013, expanding the range of treatments for extensive knee cartilage damage. Initially, the periosteum was used for the fixation of cultured cartilage, but since February 2019, the introduction of collagen membranes has shortened surgery time and simplified the procedure. We report a case where we used the knotless suture bridge technique for a more straightforward and secure fixation with a collagen membrane. The patient was a 61-year-old male who experienced right knee pain a year earlier when stepping downstairs. Conservative treatment at a local hospital was ineffective, and he was referred to our department. At the initial examination, the right knee had an extension of -5° and a flexion of 130°. A simple X-ray of the right knee showed osteosclerosis with a translucent bone image at the medial femoral condyle. Weight-bearing full-length X-ray of the lower limb showed a femorotibial angle (FTA) of 186°, a hip-knee-ankle (HKA) angle of 12.5° varus, a percentage of mechanical axis (%MA) of 15%, and a medial proximal tibial angle (MPTA) of 78°, indicating a significant varus deformity. CT and MRI revealed a cartilage defect of 36 mm in length and 16 mm in width and a bone defect with a maximum depth of 15 mm at the medial femoral condyle. The patient underwent surgery for a traumatic cartilage defect of the medial femoral condyle. For the bone defect, autologous bone grafting was performed, and for the cartilage defect, ACI was done. The ACI involved fixation with a collagen membrane using 1.3 mm suture tape and BC PushLock anchor (Arthrex, Naples, Florida, United States) in a knotless suture bridge technique. Additionally, hybrid closed-wedge high tibial osteotomy (HCWHTO) was performed for alignment correction. At eight months post surgery, MRI proton density sagittal images confirmed the joint surface by the cartilage layer, and the Modified Outerbridge Cartilage Repair Assessment (MOCART) score was 80. At 12 months post surgery, the Japanese version of the Knee Injury and Osteoarthritis Outcome Score (J-KOOS) improved from 46.43 to 82.14 for symptoms, 58.33 to 83.33 for pain, 95.59 to 100 for activities of daily living (ADL), 45 to 75 for sports, and 68.75 to 87.50 for quality of life (QOL). X-rays showed an FTA of 173°, an HKA of 0°, and a %MA of 58%, indicating a favorable course. The knotless suture bridge technique for collagen membrane fixation during ACI is considered a convenient and time-saving method.

## Introduction

Autologous chondrocyte implantation (ACI) has been reported to yield favorable outcomes in the treatment of knee joint cartilage injuries [1]. In Japan, since April 2013, the second-generation ACI, JACC® (Japan Tissue Engineering, Japan), has been covered by insurance, broadening the treatment options for extensive knee joint cartilage injuries [2]. Initially, the periosteum was used as the cover membrane for the cultured cartilage, but there were scattered reports of poor outcomes due to the poor quality of the periosteum [3]. Since February 2019, the introduction of collagen membranes in Japan has been reported to yield good results, including reduced hypertrophy of the transplanted cartilage [4]. The original method for suturing the JACC® involved a pull-out technique, which, due to its complexity, led to reports of suturing using soft anchors [5]. We report a case where we aimed for further simplification and improved fixation in the treatment of traumatic knee cartilage defects using JACC®, employing the knotless suture bridge method for fixing the collagen membrane, and achieved favorable short-term outcomes.

## Case presentation

A 61-year-old male visited our hospital with the chief complaint of right knee pain. He first experienced this pain in his right knee joint when stepping downstairs. Despite undergoing conservative treatment at a local clinic, the pain worsened during walking and made it difficult to play golf, leading to a referral to our department one year after the injury. There was nothing notable in his medical history. He reported significant right knee pain while walking and a catching sensation during automatic flexion extension. Tenderness was observed in the medial joint space of the right knee, and patellar percussion was positive. The range of motion for the right knee was -15° extension and 130° flexion, and for the left knee, 0° extension and 140° flexion, indicating a mild limitation in mobility. The Japanese Orthopaedic Association (JOA) score was 75 for the right knee and 100 for the left knee. The Japanese version of the Knee Injury and Osteoarthritis Outcome Score (J-KOOS) was 46.43 for symptoms, 58.33 for pain, 95.59 for activities of daily living (ADL), 45 for sports, and 68.75 for quality of life (QOL).

**Figure 1:**
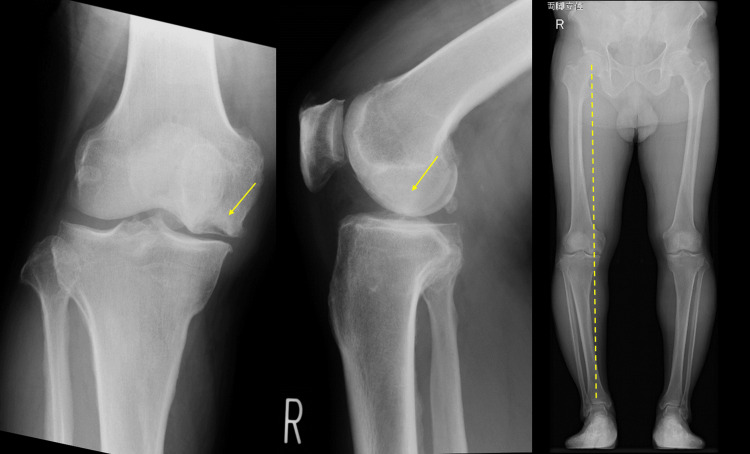
Preoperative radiograph of the right knee The arrows indicate bone and cartilage defects. The dashed line is the Mikulicz line.

**Figure 2:**
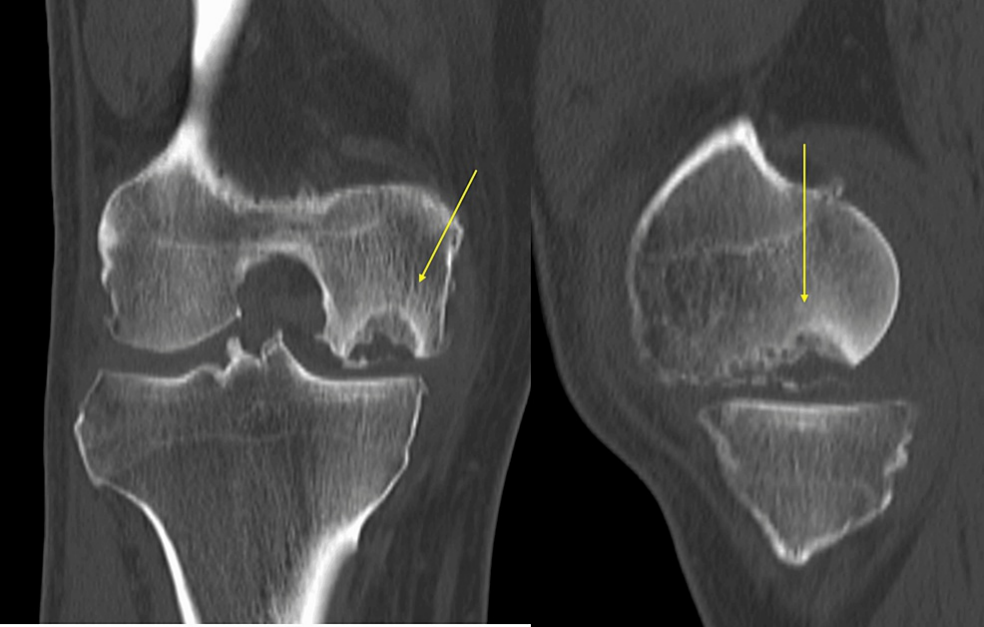
Preoperative CT scan

**Figure 3:**
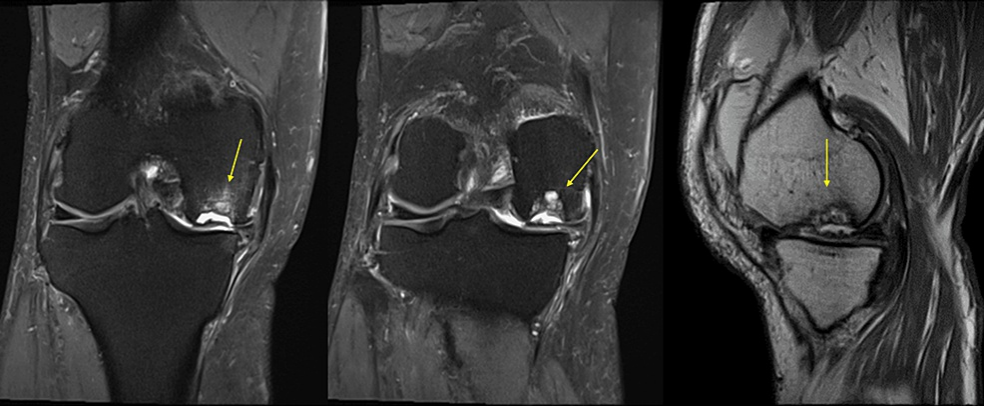
Preoperative MRI The arrows indicate bone and cartilage defects with bone marrow edema.

**Figure 4:**
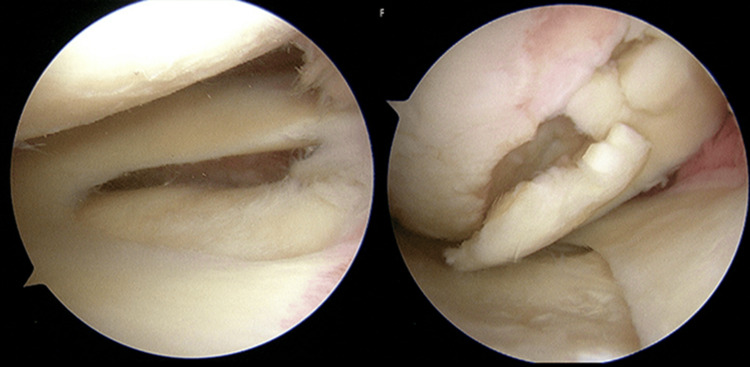
Initial arthroscopic findings

Imaging findings on simple X-rays (anterior and lateral views) showed a translucent bone image suggesting bone defects with partial osteosclerosis at the medial femoral condyle and concavity of the joint surface (Figure 1). Standing full-length leg anterior view revealed a hip-knee-ankle (HKA) angle of 12.5° varus (left 6.3° varus), a femorotibial angle (FTA) of 186° (left 180°), and a percentage of mechanical axis (%MA) of 15% (left 30%), indicating a varus deformity in the right knee (Figure 2). CT scans showed a bone defect at the joint surface of the medial femoral condyle measuring 36 mm in length and 16 mm in width and with a maximum depth of 15 mm (Figure 3). MRI proton density images showed uniform high signals at the bone defect, suggesting bone marrow edema at the medial femoral condyle (Figure 4). The diagnosis was traumatic bone and cartilage defect of the medial femoral condyle, for which bone grafting, ACI, and periarticular osteotomy were performed.

**Figure 5:**
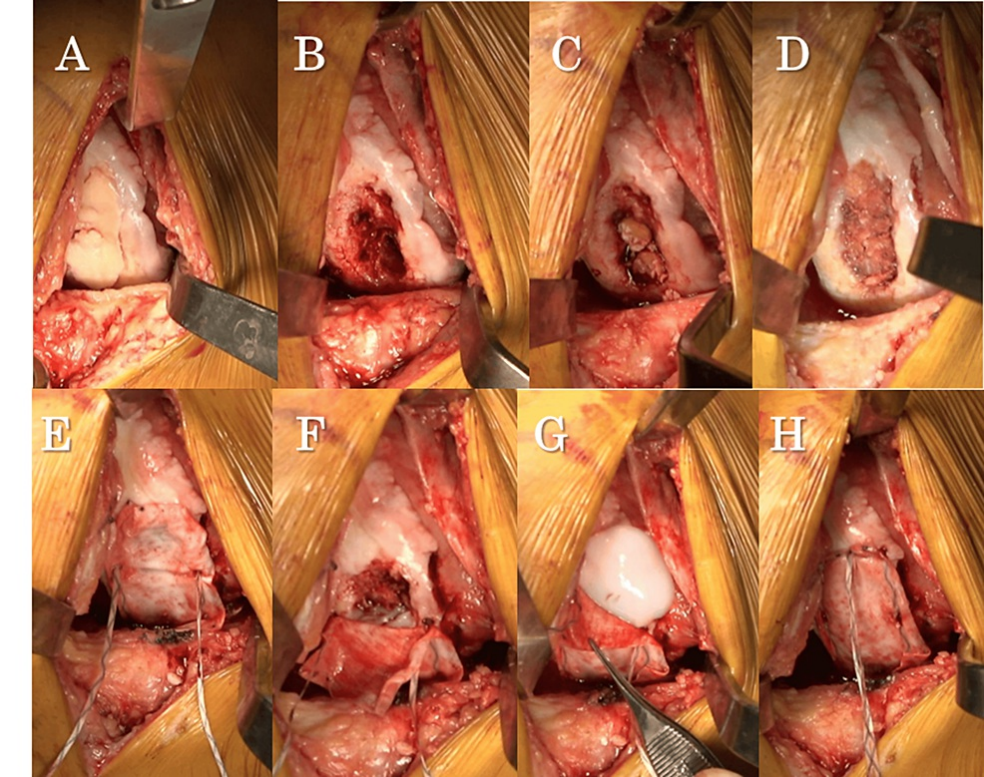
(A-H) Surgical findings of autologous chondrocyte implantation using the knotless suture bridge technique

**Figure 6:**
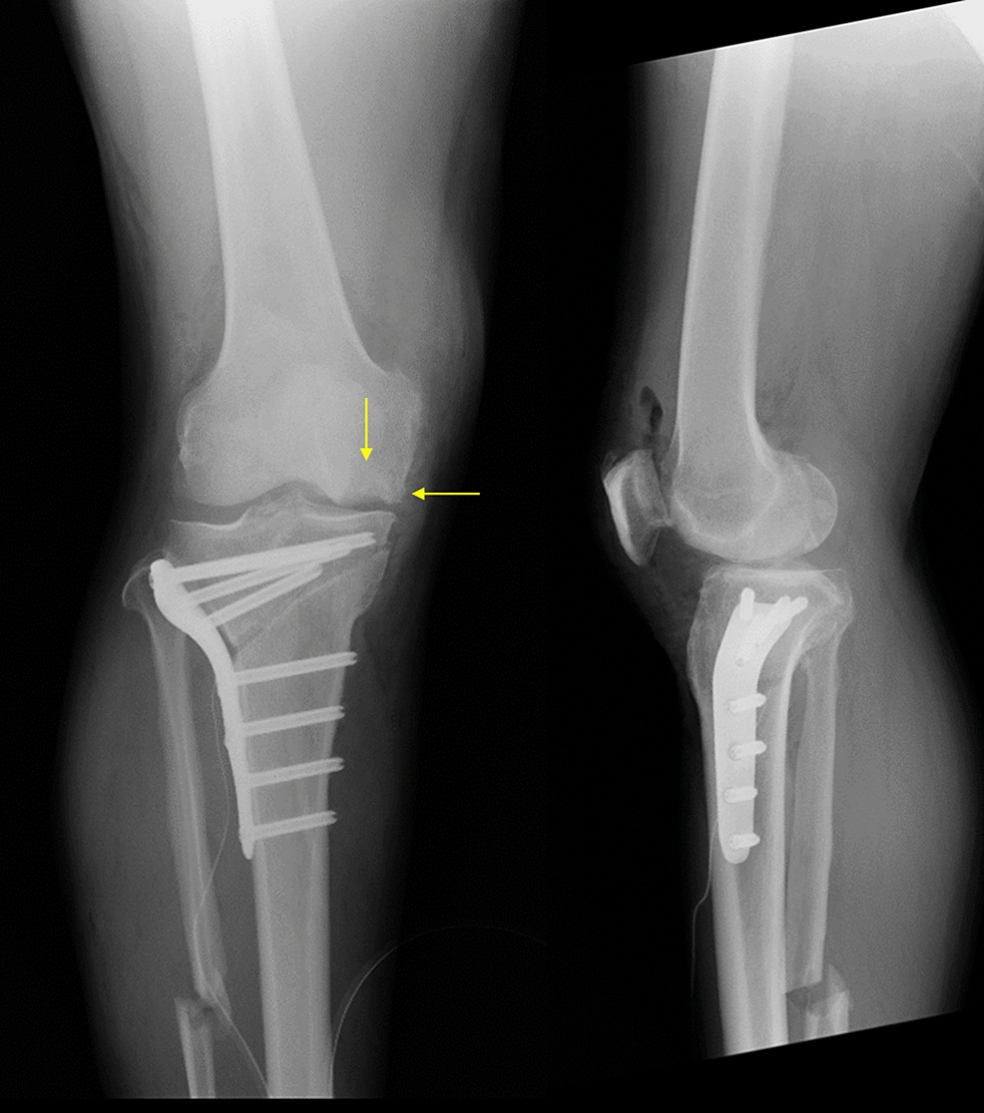
Postoperative radiograph of the right knee

During the initial surgery, arthroscopic harvesting of autologous cultured cartilage was carried out. The cartilage at the medial femoral condyle was detached, with some parts being loose (Figure 5). Cartilage was harvested from the non-weight-bearing part of the lateral femoral condyle. Four weeks after the first surgery, bone grafting, autologous cultured cartilage implantation, and periarticular osteotomy were performed. The knee joint was accessed via a subvastus approach. The detached cartilage at the medial femoral condyle was excised, and the bone defect was thoroughly curetted and freshened until the cancellous bone was exposed (Figure 5A, Figure 5B). Bone was harvested in a columnar fashion from the non-weight-bearing part of the lateral femoral condyle using Osteochondral Autologous Transplantation (OATS^Ⓡ^), and bone grafting was performed in the bone defect (Figure 5C), and cancellous bone was packed into the defect (Figure 5D). After bone grafting, a collagen membrane larger than the cartilage defect was shaped and fixed by inserting and securing it along with Arthrex's BioComposite™ (BC) PushLock^Ⓡ^ 2.9 mm anchor (Arthrex, Naples, Florida, United States) and 1.3 mm suture tape drilled into the normal cartilage at the center of the lower edge of the cartilage defect. Suture tape was loaded into BC PushLock^Ⓡ^ after drilling on the inner and outer edges below the cartilage defect to create a suture bridge (Figure 5E). After creating the suture bridge up to the central inner and outer edges of the cartilage defect, JACC^Ⓡ^ was transplanted into the bag-shaped defect formed by the collagen membrane (Figure 5F). A suture bridge was created up to the upper inner and outer edges of the cartilage defect, further transplanting JACC^Ⓡ^, and finally, the suture tape from the inner and outer sides was passed through the BC PushLock^Ⓡ^ at the upper central edge of the cartilage defect to fix the collagen membrane, completing the ACI (Figure 5G, Figure 5H). Lastly, alignment correction was performed with a hybrid closed-wedge high tibial osteotomy (HCWHTO) (Figure 6).

**Figure 7:**
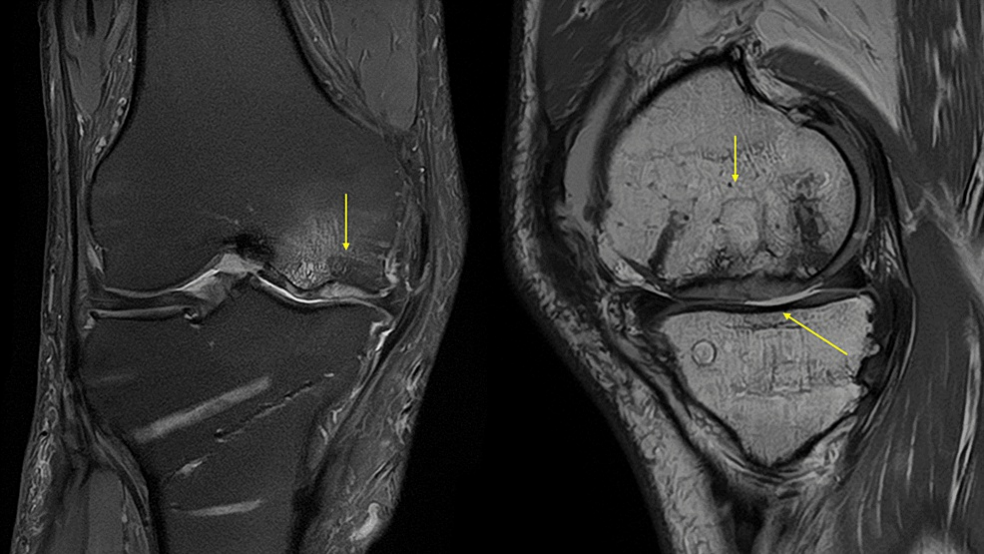
MRI at one year postoperatively The arrows indicate lesions with autologous chondrocyte implantation.

**Figure 8:**
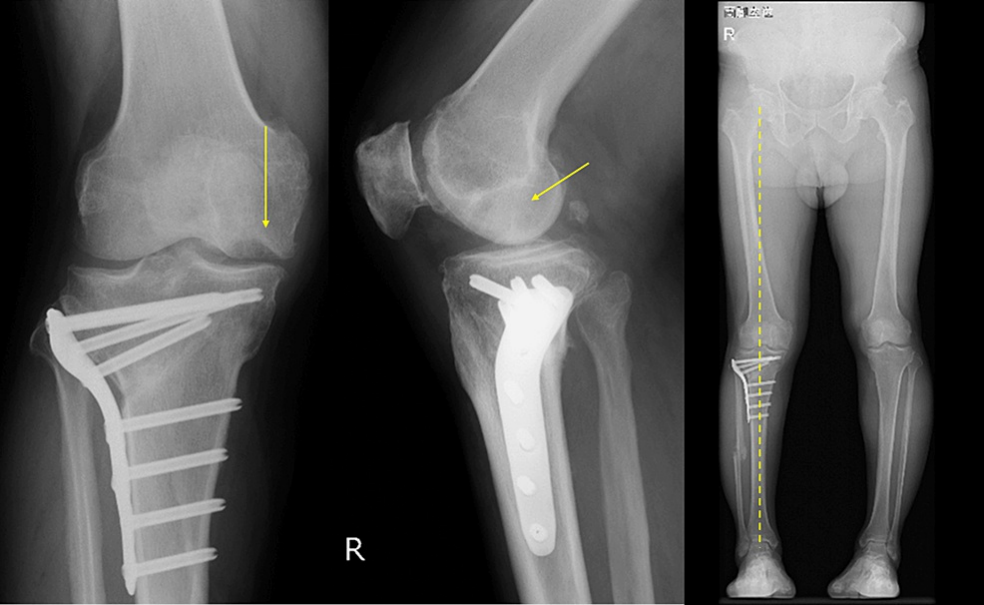
Radiograph of the right knee at one year postoperatively The arrows indicate bone and cartilage defects. The dashed line is the Mikulicz line.

Postoperatively, rehabilitation began with range of motion exercises starting one week after surgery, and 1/3 partial weight bearing (PWB) commenced in the third week. Full weight bearing was allowed at six weeks, and the patient was discharged home. Bone healing was observed six months post surgery. Eight months after the operation, MRI showed a well-formed thickness in the transplanted area with a continuous cartilage layer, and the Modified Outerbridge Cartilage Repair Assessment (MOCART) score was 80 points (Figure 7). One year after the cartilage surgery, the range of motion improved to -5° extension and 135° flexion. The JOA score improved from 75 preoperatively to 90. The J-KOOS showed improvement in symptoms from 46.43 to 82.14, in pain from 58.33 to 83.33, in activities of daily living (ADL) from 95.59 to 100, in sports from 45 to 75, and in quality of life (QOL) from 68.75 to 87.50. Simple X-ray results showed an FTA of 173°, an HKA angle of 0°, and a %MA of 58%, indicating a favorable outcome (Figure 8).

## Discussion

In the use of JACC^Ⓡ^ for ACI, the original fixation method involved a complex pull-out technique [1,2]. This method, while effective in ensuring the membrane's press fixation to the bone and cartilage, presented challenges. Its complexity and difficulty, especially in certain lesion locations, led to the exploration of simpler techniques, such as the use of JuggerKnot^Ⓡ^ soft anchors (Zimmer Biomet, Warsaw, Indiana, United States), which reported positive outcomes [5]. Our transition to JuggerKnot^Ⓡ^ for periosteal fixation, followed by a shift to a collagen membrane, was motivated by the need for a more consistent approach and less hypertrophic response in transplanted cartilage [6]. However, this method faced challenges, as the transplanted cartilage sometimes protruded through suture gaps during knee movements.

Consequently, we focused on the knotless suture bridge technique, frequently used in rotator cuff repair surgery [7], and the false-pouch closure technique [8] in peroneal tendon dislocation. This necessitated a fixation approach with a broader contact area, leading us to the knotless suture bridge technique. The knotless suture bridge technique, commonly employed in rotator cuff repair surgery, offered a solution with its high contact area and secure fixation. In our case, the technique successfully prevented any leakage of the transplanted cartilage during surgery.

Regarding the BC PushLock^Ⓡ^ anchors used in this procedure, made of bioabsorbable poly-L-lactic acid (PLLA) and beta-tricalcium phosphate (β-TCP) material, there have been concerns about postoperative bone hole expansion. Studies in rotator cuff repair surgeries using similar absorbable anchors have shown varying results, with some cases exhibiting bone hole enlargement but no significant impact on clinical outcomes or reoperation rates. Pilge et al. have reported that in rotator cuff repair surgery using Bio-Corkscrew (Arthrex, Naples, Florida, United States), bone hole enlargement was observed in 22 out of 70 cases after more than 24 months of follow-up [9]. Lee et al. reported that there was no difference in clinical outcomes or reoperation rates with or without bone hole enlargement in rotator cuff repair surgery using absorbable anchors [10]. Tompane et al. reported that cyst formation with the use of soft anchors in Bankart repair was infrequent, but the cysts expanded over time [11]. Additionally, Ro et al. found no significant differences in the incidence of perianchor cyst formation in arthroscopic rotator cuff repair among three groups using soft anchor, bioabsorbable anchor, and PEEK anchor (Acumed, Hillsboro, Oregon, United States) [12].

In conclusion, the use of the knotless suture bridge technique in ACI for treating medial femoral condyle defects demonstrated ease of use and efficacy. While short-term outcomes were favorable, ongoing monitoring for potential long-term issues, such as bone hole enlargement, remains essential.

## Conclusions

In the autologous cultured cartilage transplantation surgery for the treatment of bone and cartilage defects in the medial femoral condyle, the knotless suture bridge technique was used to fix the collagen membrane. The clinical outcomes one-year post surgery were favorable, indicating that the knotless suture bridge method is a useful technique for fixing the cover membrane of the cultured cartilage.
